# Supporting Rural Australian Communities after Disaster: the Warrumbungle Bushfire Support Coordination Service

**DOI:** 10.1371/currents.dis.6a4ee241c389755ad6f6f1c8688c0fb5

**Published:** 2015-06-01

**Authors:** Jacqueline Coombe, Jane Rich, Angela Booth, Allison Rowlands, Lisa Mackenzie, Prasuna Reddy

**Affiliations:** Hunter Medical Research Institute (HMRI); Centre for Rural and Remote Mental Health, School of Medicine and Public Health, Faculty of Health and Medicine, University of Newcastle, Newcastle, New South Wales, Australia; Hunter Medical Research Institute (HMRI); Centre for Rural and Remote Mental Health, School of Medicine and Public Health, Faculty of Health and Medicine, University of Newcastle, Newcastle, New South Wales, Australia; Centre for Rural and Remote Mental Health, School of Medicine and Public Health, Faculty of Health and Medicine, University of Newcastle, Newcastle, New South Wales, Australia; New South Wales Ministry for Police and Emergency Services, Sudney, New South Wales, Australia; Hunter Medical Research Institute (HMRI); Centre for Rural and Remote Mental Health, School of Medicine and Public Health, Faculty of Health and Medicine, University of Newcastle, Newcastle, New South Wales, Australia; School of Medicine and Public Health, Faculty of Health and Medicine, University of Newcastle, Newcastle, New South Wales, Australia

## Abstract

Aim: Natural disasters inflict significant trauma upon the individuals and communities in which they occur. In order to gain an understanding of the role of community-based disaster recovery support services in the post-disaster environment, we assessed the acceptability and perceived effectiveness of the Warrumbungle Bushfire Support Coordination Service (BSCS) implemented in response to the January 2013 bushfires in the Warrumbungle Shire, New South Wales, Australia.

Method: A mixed-methods approach was taken to explore the perspectives of former BSCS users and key stakeholders involved with the service. A survey was distributed to former services users (in both paper and online modalities) and included closed and open-ended questions. Semi-structured interviews were conducted with key stakeholders (face to face or via telephone).

Results: A total of 14 former BSCS users and six key stakeholders participated in the research. Almost half of the former service users had accessed the BSCS for more than six months. Regardless of the duration of their use of the service, most reported that the decision to use the service stemmed from the need for ‘help’. The majority of former service users were satisfied with the support provided by the BSCS and would recommend the service to others. Although most indicated that the BSCS informed them about where to get support, just over half were confident that they could access appropriate recovery services without the BSCS. Key themes arising from the former service use surveys were connectedness and support, whilst key themes in the interviews with key stakeholders were connectedness and the operation of the service. Both former service users and key stakeholders reported that the BSCS played an important role in facilitating community connectedness in the post-disaster period. Key stakeholders also identified challenges for the BSCS, including finding an appropriate agency and location to oversee the service and made suggestions about sustainability.

Conclusion: On the whole, the BSCS was perceived by former service users and key stakeholders as acceptable and effective. To develop a better understanding of the role of community-based disaster recovery support services, there is a need for more timely, rigorous and representative evaluation of disaster support services like the BSCS. Recommendations are made for the planning and development of future disaster support services. Key words: bushfires, natural disaster, Australia, disaster recovery support service, rural and remote, communities

## Introduction

Australia experienced extreme heatwaves and record breaking temperatures over the 2012/2013 summer period (December-February). Across the country, the average maximum temperatures were the highest on record 1 with January 2013 being the hottest month recorded since 1972. This widespread heatwave created conditions conducive to catastrophic bushfires 2.

During these extreme conditions, on Sunday 13^th^January 2013, the Wambelong Bushfire burnt out of control in the Warrumbungle National Park, in the State of New South Wales (NSW), Australia. Situated in the Warrumbungle Shire approximately 5-6 hours north-west from the state capital of Sydney, the Warrumbungle National Park and Siding Spring Observatory have been popular tourist destinations 3. The Warrumbungle Shire has a population of 9832 people 4 and encompasses the surrounding towns of Coonabarabran, Coolah, Dunedoo, Mendooran, Baradine and Binnaway 3. The Wambelong Bushfire continued to burn for more than a week, threatening the Warrumbungle communities and their attractions, before it was finally contained on Monday 21^st^January 5. By the time the bushfire was brought under control it had destroyed more than 53,000 hectares of land, 51 homes and 113 outbuildings, livestock and farm machinery 6, as well as causing significant damage to the Warrumbungle National Park and the Siding Spring Observatory infrastructure.

It is well documented that natural disasters inflict significant trauma upon communities. Individuals living in affected areas may face major personal and community wide challenges throughout the recovery process 7. Recognising that the recovering community may require support to manage challenges arising from the January 2013 bushfires, the Warrumbungle Bushfire Support Coordination Service (BSCS) was implemented in the Warrumbungle Shire. The BSCS was conceptualised within the Disaster Welfare Services Branch of the Ministry for Police and Emergency Services (MPES DWS), and discussed with Warrumbungle Shire Council in the days immediately following the fire. Consultation with NSW Treasury and the Emergency Management Australia Division of the Attorney-General’s Department gained support for the proposal. The NSW and Commonwealth governments, through the Natural Disaster Relief and Recovery Arrangements, jointly funded the service, with in-kind support from the Warrumbungle Shire Council and NSW Health.

The BSCS was an outreach service that provided an individualised and relationship-based service to adversely affected households. The aim of the coordinated service was to support individuals and facilitate their recovery from the impact of the bushfire, and strengthen their capacity over time. Utilising a person-centred, strengths-based and solution-focused approach, the BSCS support workers provided a point of contact to help households engage with services, provide support, assist in seeking financial assistance or information, navigate the range of services, grants and loans that were available, and co-ordinate with appropriate agencies for immediate needs. Service delivery was locally-driven and coordinated with support from Local, State and Commonwealth Governments. The service was linked into the broader recovery processes of the community, including being represented on the Recovery Committee and its Human Services Sub-Committee. This facilitated a ground-up approach to providing the community service. The BSCS was established as a temporary recovery service that commenced in February 2013 and functioned within the Warrumbungle Shire for approximately six months. The service ceased operation in early August 2013, after consultation between MPES DWS and the BSCS coordinator.

In the Australian context, there have been few evaluations of government initiated recovery and support interventions following a natural disaster. One of these few was the evaluation of the Victorian Bushfire Case Management Service conducted in 2009 by Urbis 7 . This study found that using a case management model for community disaster recovery proved beneficial for those involved, including clients, staff and stakeholders. The results demonstrated high client satisfaction with this model. In particular, the frequency of ‘client to case manager’ contact was the biggest driver of satisfaction, followed by case managers advocating on behalf of their clients. These results suggest that communication and a proactive outreach to client groups enables responsiveness and support to take place.

While recovery services aim to support individuals through these challenging times, there is little research investigating the degree to which these services help or hinder the recovery process. Although past evaluations of disaster recovery support services suggest that successful interventions promote social connectedness and community development, there is a lack of substantial evidence about which intervention strategies result in positive outcomes 7. In the absence of rigorous evaluation design and thus evidence of effectiveness, service user and stakeholder perspectives of a service (such as the BSCS) may provide useful guidance as to how future services could be improved. Additionally, the role of government assistance in post-disaster intervention is not well understood, despite research which indicates the necessity of governments to be ‘evidence-informed’ in implementing post-disaster community services 7 . Given that natural disasters can devastate communities, there is a need to evaluate government services that are implemented following a natural disaster 13 in order to enhance their effectiveness. More knowledge is urgently needed in order to guide disaster preparedness response policies 15.

Recognising this knowledge gap, the evaluation of the Warrumbungle BSCS aimed to ascertain the acceptability and perceived effectiveness of the service, and assist the further development of the NSW Government’s disaster response and recovery policy. Specifically, this investigation assessed former BSCS service users’ perceptions of whether the BSCS was responsive to their needs, whether it assisted in developing knowledge about where to seek support, and how to navigate and access recovery services when required. This study was also interested in the experiences and perceptions of the stakeholders involved in the delivery of the recovery service. The results presented in this paper comprise part of the research internally reported in an evaluation commissioned by MPES 11

## Method

**Ethics Approval**

Ethics approval was granted by the University of Newcastle Human Research Ethics Committee in October 2013.

**Evaluation Design**

A mixed methods design was used to address the evaluation aims, utilising both survey and interviews. This methodological approach is recommended for research focusing on responses to natural disasters such as bushfires 14.

**Participants and procedure**

Eligible participants comprised former Warrumbungle BSCS users and key stakeholders involved with the service. Participant recruitment commenced one year after the bushfires and continued until July 2014.

*Former service users*

Former Warrumbungle BSCS service users were identified by MPES DWS who retained all client information at the conclusion of the BSCS. Of the 82 households which used the service, approximately 78 former service users had previously indicated their consent to be contacted for matters involving their use of the service. These people were posted an invitation letter, participant information statement, paper survey and reply paid envelope. Two reminder letters, including another participant information statement and survey, were sent to the participants who had not responded to the initial mail out. These reminders were sent one and two months after the initial invitation. To facilitate completion, a web-link to the online survey (identical to the paper survey) was provided with the second reminder letters.

*Key stakeholders*

Key stakeholders from the human services sector who had worked closely with the BSCS during its term of operation were identified by MPES DWS personnel. A MPES DWS staff member compiled a mailing list of 26 potential participants and emailed each of them a participant information statement and informed consent form. Up to two reminder emails were sent. Participants were asked to complete an informed consent form, including their contact details and return it to the research team. They were advised that the researchers would be in touch to organise a mutually convenient time and day for an interview. Interviews with key stakeholders were conducted face-to-face or via telephone by a member of the research team and audiotaped to facilitate transcription and analysis.

**Measures**

Study questionnaires and interview schedules were developed and reviewed by the evaluation team, drawing on past disaster support services evaluation frameworks 16.

*Former service users questionnaire*

Identical paper and online surveys, programed with Survey Monkey 21, were utilised. The survey contained a series of closed and open ended questions assessing participant characteristics, level of involvement with the Warrumbungle BSCS, and perceptions of the service.

*Key stakeholder interviews*

A semi-structured interview (designed for either face-to-face or telephone administration) assessed key stakeholder level of involvement with the BSCS, their perceptions of the service and any recommendations for future disaster-recovery services.

**Data Analysis**

IBM SPSS Statistics 17 was used to produce descriptive statistics based on the quantitative data from the former service user surveys, and Stata 11.2 20 was used to estimate 95% confidence intervals (CIs). Basic qualitative analysis on the open-ended questions was conducted, with the short responses coded thematically using NVivo 19. Key stakeholder transcripts were also uploaded to NVivo to facilitate analysis. A thematic analysis approach as described by Braun and Clarke 10 was utilised to identify key themes which emerged from the interview transcripts. This analysis approach involved familiarisation with the data, generating initial codes, searching for themes, reviewing themes and defining and naming themes 10.

## Results

**Consent rates**

Of the 78 former users who were invited to participate in the study, 14 (response rate of 18%) returned completed surveys. Six of the 26 (response rate of 23%) key stakeholders consented to participate in an interview.

**Former service users**

The 14 participants were a mean age of 60 years (range 43-73 years), and comprised 9 males and 5 females. All had the same residential postcode. All participants indicated that they had experienced personal loss or property damage in the January 2013 bushfire, almost half had used the BSCS for six or more months, and many indicated that they had first heard about the service though a friend (Table 1).

**Table 1. Service usage of former service users (n = 14):** 

Service Usage		N (%)
For how long did you use the service?	Up to two months	4 (29)
	Three to five months	4 (29)
	Six months or more	6 (43)
		
How did you first hear about the service?	Through a friend	5 (36)
	Through a family member	2 (14)
	Through the media	2 (14)
	Other	5 (36)

Reasons for the decision to use the BSCS were generally related to ‘help’. Help with paperwork, financial assistance, personal support or a combination of these were all reported in response to the open ended question ‘what made you decide to use the service?’. All of the respondents, except one, indicated that the BSCS had successfully informed them about where to get support (Table 2). Of the 14 participants, only seven said they were now confident accessing appropriate support services without the help of the BSCS.

**Table 2. Quantitative survey results former service users:** 

Survey Questions		N* (%, 95% CI)
Did you meet any new people through the BSCS, or renew an old friendship?	Yes	8 (62, 32-86)
	No	5 (38, 14-68)
If yes, are you still in contact with these people?	Yes	7 (88, 47-100)
	No	1 (13, 0-53)
Did the BSCS help to improve the sense of community in the Warrumbungle Shire since the bushfires?	Yes	9 (69, 39-91)
	No	4 (31, 9-61)
Did the BSCS inform you about where to get support?	Yes	13 (93, 66-100)
	No	1 (7.1, 0-34)
Do you now feel confident that you could access appropriate recovery services without the BSCS?	Yes	7 (54, 25-81)
	No	6 (46, 19-75)
Do you feel that the services provided by the BSCS were responsive to your individual needs?	Yes	12 (92, 64-100)
	No	1 (7.7, 0-36)
Did you feel respected as an individual in your interactions with the BSCS?	Yes	14 (100, 77-100)
	No	0 (0, 0-23)
In your opinion, was the BSCS available to you when you needed it?	Yes	12 (86, 57-98)
	No	2 (14, 1.8-43)
Did you recommend the service to a family member or friend?	Yes	11 (79, 49-95)
	No	3 (21, 4.7-51)
On the whole, did you feel satisfied with the support provided by the BSCS?	Yes	10 (83, 52-98)
	No	2 (17, 2.1-48)
*Participant N may not add to total sample size due to missing responses to some questions		

Overall, two key themes arose from the analysis of the qualitative survey data; connectedness and support. These themes are defined in Table 3. The quantitative data that aligned with these qualitative themes are also presented below.

**Table 3. Qualitative survey results former service users:** 

Theme	Definition	Example Quote
Connectedness	Social connections to the local community or connection to the local community	“Gave people a sense of belonging with considerable community involvement and commitment to ensuring recovery”.
Support	Support between service user and BSCS worker, interpersonal skills	“Calmly taking me through all the assistance that was available without being officious and intrusive”

*Connectedness*

Through the delivery of this bushfire recovery service the BSCS aimed to enhance feelings of connectedness and belonging. Given the great loss and displacement experienced by many, workers demonstrated strong empathy and support early and consistently through the recovery process. When service users were asked if the BSCS improved their sense of community belonging, 69% (95% CI: 39%, 91%) of respondents said that the service did increase community cohesiveness in the Warrumbungle Shire. The survey also asked if the service introduced new people into their lives or if they renewed friendships as a result of using the BSCS. Over half (62%; 95% CI: 32%, 86%) of service users surveyed said that they had either met someone new or renewed an old friendship through the BSCS. Of these, 88% (95% CI: 47%, 100%) were still in contact with those people at the time of the survey. These findings are encouraging and suggest that for this group, the BSCS was able to contribute to their sense of community connectedness and cohesiveness.

The qualitative data from the survey further explores community connectedness. Respondents were asked to expand on how they felt the BSCS contributed to an increased sense of community. Participants said that the *“[BSCS] gave people a sense of belonging with considerable community involvement and commitment to ensuring recovery”* and *“by giving people options and choices to help improve their situation, showing people that they are not alone”*. Participants particularly felt that the way that the service introduced them to others going through similar experiences was also helpful in building community connectedness. One participant stated the BSCS provided assistance *“through introductions to other people in similar situations…listening and advising”.* Additionally, the BSCS introduced them to local support agencies *“by providing linkages between local charities and services and ready to access to them”.* One participant suggested that it was not the BSCS, but the fire itself, that contributed to the increased sense of community.

*Support*

Support was a key theme highlighted throughout the survey responses. Participants were asked specific questions about the role of the BSCS workers and how their role assisted in recovery. From the quantitative findings, 93% (95% CI: 66%, 100%) of respondents said that the BSCS informed them about where to get support, and that the service was responsive to their individual needs (Table 2). Respondents also acknowledged the role of the worker in this process, with 83% (95% CI: 52%, 98%) of service users feeling satisfied and supported by the BSCS, 86% (95% CI: 57%, 98%) of respondents felt that the service was available when they needed it and all service users agreed with the statement that they felt respected as an individual during their interactions with the BSCS.

Elements of respect and genuine care were also derived from the qualitative comments. Participants were asked open ended questions regarding the role of the worker and how this relationship impacted their experience of recovery. Participants described this relationship as *“gentle, caring support, information sharing, material support, incredible empathy”. *While other participants described practical aspects including *“we saw [worker] over several issues: financial aid, psychological assistance, we needed it so bad, his guidance”. *Participants described the value in having someone to listen to them, guide them through the recovery process and negotiate the system. Participants described how the workers *“help with direction, support and contacts”. *Participants also stated that they received information from workers about *“what forms to fill out” *and *“what financial help was available”.*

**Key Stakeholders**

Six key stakeholders consented to participate in an interview about their experience with the BSCS. All of these participants were female. Overall, the stakeholders indicated that they were satisfied with their involvement in the service. From the qualitative interviews two major themes emerged: connectedness and operation of service (Table 4).

**Table 4. Qualitative findings key stakeholder interviews:** 

Theme	Definition	Example Quote
Connectedness	Within community and other agencies/services	“I think, [the BSCS did] open up the potential for partnerships and information sharing between the local services and more regional services.”
Operation of Service	Timeline,availability, operational and logistical matters.	“Overall from where I sit it was a privilege to be involved in a service like this and it did give us another perspective and another set of skills and we were very well resourced to respond. So overall I was pleased that we were able to support the community. I think also what the staff would say there was it raised our profile in the community considerably.”

*Connectedness*

Many stakeholders said that improved connectedness was noticeable from the implementation of the service, particularly between the agencies involved with BSCS, between agencies in the region, and between agencies and the community in general.

“Well, I think it [the BSCS] helped with the connection of the service system, like the service system certainly connected a lot more. That's probably what it did do well. It made us get together and talk for sure, even though some key people were missing - those that attended, yeah.”

Stakeholder 4

“I think, [the BSCS did] open up the potential for partnerships and information sharing between the local services and more regional services.”

Stakeholder 2

Stakeholders indicated that accessible and timely information was an important aspect of this increasing connectedness.

“So the biggest impact that I think we had on the clients in general as they came, was a bit of an open door policy. Come in, have a chat, debrief, get things validated, someone to update, someone to share that whole validation processing thing.”

Stakeholder 4

*Operation of service*

Daily management, service operational matters and service logistics were important aspects of the BSCS experience. During the interviews stakeholders highlighted key aspects which worked well within the service. Alternative or future changes that could take place to enhance the operational side of delivering the service were also discussed. Often stakeholder comments indicated that the BSCS served the community in a positive way and that the agencies involved were happy to be involved with the service.

“Well one thing is if it happened again - hopefully it won't - I would be very happy, and my colleagues would be, to be involved in it again. I thought it was a good model for what we had to do at the time, never having had it done before.”

Stakeholder 1

“Well I guess the first thing I would imagine would be how timely it was. The second thing is it's absolutely essential in those sort of groups to have the right people and [first name] did a very good job. Then I guess, as we've been talking about, just that communication, making sure that everyone's in the loop and knows exactly what's going on, well and truly.”

Stakeholder 6

There were also a number of comments made about the practicalities of running the BSCS within the community. These were both tangible, such as the location of the service, and intangible, such as the working relationship between the agencies and people involved in administering the service. The working relationship between the BSCS workers on the ground was discussed by some of the participants, who articulated the difficulties inherent in working with people from multiple theoretical backgrounds and models of service.

This was particularly evident in the communication between the collaborating agencies. This is illustrated by one of the stakeholders who commented about the transparency of the appointment of the BSCS Coordinator role.

“It's a really hard one because they had to get someone, they had to get up and running and they had to do it now and MPES did it and they selected someone who was very appropriate. But I just wonder if the rest of the service sector felt like there was transparency in how that person came on board.”

Stakeholder 5

The apparent lack of transparency between the agencies was a significant issue raised by a number of the participants. Hence, suggestions and comments about how transparency could be better addressed in the future were provided by some of the stakeholders who also acknowledged the challenges inherent in working within small communities.

“So I think the key thing is to try and…and I know that's very hard, especially in a small community where everybody knows everybody and it can get quite tricky. But choosing key people that have really good solid relationships in the community and that's very hard to find in setting up a very quick service after a disaster.”

Stakeholder 4

More tangible practicalities of the administration of the service were also commented upon. These included the physical location of the service as problematic and the timeframe within which the service functioned.

## Discussion

The evaluation of the Warrumbungle BSCS aimed to ascertain the acceptability, feasibility and perceived effectiveness of the service. Overall, both the former service users and the key stakeholders were satisfied with the support the BSCS provided. Most of the former service users felt that the BSCS was responsive to their individual needs and all participants reported that they felt respected as an individual in their interactions with the BSCS. The majority of the participants also referred the service on to a family member or friend, which suggests that the service was individually beneficial.

Interviews with key stakeholders revealed that the implementation of the BSCS in the community was conducted in a timely manner. Both the former service users and the key stakeholders indicated that a positive outcome of the BSCS was an increase in communication in the community. For key stakeholders this communication constituted increased interaction between themselves and agencies involved with the BSCS, and agencies in the area and the wider community. The BSCS assisted in linking local charities and services and was reported by the services users as beneficial to their recovery. Research has shown that these networks, such as those facilitated by social capital, are particularly important for community recovery post-disaster 22 ^,^ 23

Despite these positive outcomes of the BSCS, there were a number of comments made around challenges with or improvements to the service. Some stakeholders commented on issues related to the administration of the service in the community. The relationship between some of the agencies working with the BSCS, including the approach taken to recovery, the location of the service and issues with how the BSCS was terminated, were discussed as service limitations.

Additionally, although most former service users indicated that the BSCS had successfully informed them about where to get support, only a minority reported that they felt confident accessing the appropriate recovery services without the BSCS. This suggests that the BSCS did not necessarily increase confidence in accessing services once the formal support of the BSCS ceased. Thus, any temporary service like the BSCS that is established to assist disaster recovery needs to have clear sustainability objectives, such as how the service can provide users with the skills to access the support they require after the immediate disaster recovery support is terminated.

## Limitations

These findings should be considered within the context of research conducted in a post-disaster environment, and the associated challenges of this. In particular, the ethical implications of undertaking research on a population of people who may be experiencing the after effects of a disaster, including psychological, social or economic difficulties, must be taken into consideration 18. Additionally, the unpredictable nature of disasters does not lend itself to the development of well-considered, tailored responses, given the often rigid nature of research protocols 9. Research in a post-disaster setting needs to be flexible, holistic and hold some benefit for those to be involved. Pre-existing collaborations between research groups and disaster recovery organisations prior to disasters may mediate some of these impacts by allowing, at least, for the framework to be predefined, and research processes to be clarified 9. Importantly, the needs of the individual and the community must be balanced with the aims and outcomes of the research 8 ^,^ 12 which may have important implications for future disaster recovery processes.

The overall low response rates and high proportion of females in the sample may have been due to a number of factors. Recruitment for this type of research is difficult in rural areas where distance, isolation and the number of people eligible for participation compared with urban regions comes into play. Specific to our study, low recruitment rates may also be a result of commencing data collection one year post-disaster. Some former service user invitations were returned as unknown addresses and some key stakeholders had either left the jobs they were working in during the term of the BSCS, or were unable to be contacted during the evaluation recruitment period. Additionally, those who were available for interview were all women, which may have produced a gender bias we are unaware of. This delay in recruitment may have also impacted on participant recall, and therefore the depth of data collected. The resulting small sample meant quantitative findings were qualified by wide confidence intervals. However, consistency between quantitative responses and themes arising from the qualitative data suggests the mixed method approach to assessing former service user perspectives may have helped to enrich the findings that would have been reported using either method alone 14.

Given the limitations of the evaluation design, and that the survey instrument did not utilise a measure of actual recovery or severity of loss, we are unable to determine whether the BSCS was successful in assisting the community recover from disaster. Our sample may also be biased, in that only those people who experienced mostly positive experiences with the service chose to respond. Additionally, as the interview participants were all female, this may have produced a gender bias in responses. In combination with our small sample size, we are unable to make conclusive statements about the success, and thus potential positive outcomes, of the BSCS. However, our research does shed light on the role post-disaster support services play from the perspectives of clients and key stakeholders, whilst highlighting the need for further well designed research to explore effectiveness of similar services.

Additionally, in analysing data from both the former service user surveys and key stakeholder interviews, it became apparent that at times the participants were talking about services other than the BSCS which operated in the immediate recovery period. It is likely that in times of disaster, people impacted by the event may not distinguish between recovery services. While this is understandable, future evaluations would benefit from added clarity about the service of interest. Although future evaluations would benefit from more timely administration and a larger sample of respondents, the findings from this mixed methods approach are useful for guiding improvements in the implementation, management and termination of support services like the BSCS.

## Lessons learnt and considerations for the future

This evaluation has provided a greater understanding of the positive and negative aspects of the BSCS. This information may assist in the development of a revised service framework to better meet the needs of future clients of similar services. There were a number of considerations for the establishment of future services like the BSCS arising from this research. The importance of selecting agencies with a compatible theoretical background and practice approach was highlighted. This inherent compatibility is important for effective communication and collaboration between all participating services and their ability to disseminate their support to the community appropriately. Additionally, providing informative communication with key stakeholders will benefit the community because they will in turn provide this information to community members. The physical location of the service in the community should also be carefully chosen, to ensure wider use of services. The most neutral, culturally appropriate and accessible site should be utilised. These may include local spaces such as the library or town hall.

Future evaluations of disaster recovery services, while needing to be mindful of not placing undue burden on impacted communities, might consider conducting their investigation closer to the event in the hope of achieving higher participant numbers with potentially more accurate recall. Additionally, people affected by disaster do not always differentiate between which organisation is responsible for, and is offering what service. Thus, it is important to be descriptive and specific in data collection methods about the particular service being evaluated. Involving research teams in the implementation phase of a support service would fully optimise evaluation opportunities.

## Conclusion

Overall, both former service users and key stakeholders were satisfied with their interactions and experiences with the Warrumbungle BSCS. Former service users indicated that they sought support from the BSCS for administration, financial and personal support. However, not all of the former service users reported that they felt confident seeking out these services without the aid of the BSCS. Additionally, while key stakeholders reported positive outcomes of the BSCS and their involvement with the service, there were a number of challenges and suggested improvements put forward. The survey and interviews were a valuable way to gain insight into the way the service functioned within the community from people who regularly interacted with it to improve services like the BSCS. While keeping in mind the broader disaster context and in particular, the issue of needs versus resources, improvements in the implementation, management and termination of disaster recovery support services like the BSCS can occur to ensure the needs of the community are appropriately met.

## Competing Interests

All authors were employed by the University of Newcastle, except one author who was employed by the government department overseeing high level management of the BSCS. This author had no direct involvement in collection or analysis of data.
